# Development and Validation of a Malaysian Blood Donor’s Satisfaction Questionnaire

**DOI:** 10.21315/mjms2021.28.3.8

**Published:** 2021-06-30

**Authors:** Pei Pei Tan, Chee Tao Chang, Jernih Abdul Rahman, Sabariah Mohd Noor

**Affiliations:** 1 Transfusion Medicine Department, Hospital Raja Permaisuri Bainun, Ipoh, Perak, Malaysia; 2 Regenerative Medicine Cluster, Advanced Medical and Dental Institute, Universiti Sains Malaysia, Kepala Batas, Pulau Pinang, Malaysia; 3 Clinical Research Centre, Hospital Raja Permaisuri Bainun, Ipoh, Perak, Malaysia

**Keywords:** development, validation study, surveys and questionnaires, blood donors, satisfaction, Malaysia

## Abstract

**Objective:**

To develop and validate a questionnaire which evaluates the blood donors’ satisfaction.

**Background:**

In Malaysia, blood procurement relies mainly on voluntary non-remunerated donors. Hence, it is important to ensure the satisfaction of the blood donors in order to increase retention.

**Methods:**

This study was conducted among blood donors who attended blood donation and understood the Malay language. Non-Malaysian and illiterate donors were excluded. The questionnaire was developed by the transfusion medicine team. Content validity was established by content reviewers, while face validity was examined in the cognitive debriefing stage. For the 18-item questionnaire, 90 respondents were required based on the 1:5 ratio. A retest was performed in two weeks’ time.

**Results:**

One hundred and thirty-seven participants responded in the first phase, while 103 responded after two weeks. The five domains were: technical, interpersonal, accessibility/ convenience, physical experience and overall satisfaction. The Kaiser-Meyer-Olkin (KMO) value was 0.896, with significant Bartlett’s Test of Sphericity (*P* < 0.001). The factor loadings ranged from 0.729 to 0.953. The Cronbach alpha values of the five domains ranged from 0.814 to 0.955 and the intraclass correlation coefficient ranged from 0.663 to 0.847.

**Conclusion:**

The Malaysian blood donor’s satisfaction (M-BDS) questionnaire is a reliable and valid tool suitable for the assessment of blood donor’s satisfaction in blood donation centres.

## Introduction

The need for blood and blood products is increasing annually worldwide, especially in low and middle-income countries (1). Out of the 117.4 million blood donations collected globally, 42% are collected in high-income countries, inhabited by 16% of the world’s population. Based on a sample of 1,000 people, the blood donation rate was 32.6 donations in high-income countries, 15.1 donations in upper-middle-income countries, 8.1 donations in lower-middle-income countries and 4.4 donations in low-income countries (2).

Growing demand in blood supply is a global phenomenon. In Canada, it was reported that blood demand outpaced the supply due to ageing and increase in demand (3). In Germany, the number of patients needing transfusion increased from 2005 to 2010, due to the increasing trend of ageing population (4). In China, the proportion of blood donors was lower than the world standard of 30–40 donors per 1,000 population (5). Similarly, in Japan, the number of donors in their 20s and 30s decreased every year and the estimated shortfall of blood donations is expected to increase each year (6).

Recruiting new donors and retaining existing donors were challenging. Numerous factors, including comfortable donation settings, staff attitude and professionalism may influence the non-remunerated blood donors’ intention to donate (7). A previous study in Vietnam revealed that a pleasant reception experience may make donor feels respected, thereby increased their satisfaction (8). In the United States, a study found that phlebotomists who demonstrated good interpersonal skills may reduce the incidence of adverse donor reactions (9). On the other hand, prolonged waiting time among the Irish population had led to donor’s dissatisfaction (10).

In Malaysia, blood procurement relies mainly on voluntary non-remunerated donors. According to the WHO, regular voluntary blood donation rate should be at least 5% of the population in developed countries (11). The blood donation rate was estimated at 2.0%–2.25% between the years 2011 and 2015 (12). Total number of blood transfusions had raised from 151,500 to 338,600 episodes from year 2000 to 2014 (13). The blood demand may outnumber the supply due to the ageing population, where the donors’ pool shrinks, and the recipient pool grows over the years (14). From 2008 to 2014, the repeat donors contributed more in comparison to new donors, which further indicated that decent strategies should be placed to enhance recruitment of new donors (13). Consequently, evaluation of the blood donors’ satisfaction is important to improve blood bank services and to enhance donor’s retainment

Since the blood donors’ satisfaction was widely influenced by sociocultural differences (7–10), it is logical to explore this within the local context. The Malaysian population comprised of a multi-ethnic, multicultural and multilingual society. Although there were several existing questionnaires which assessed the blood donor’s satisfaction (7–10, 15), none has addressed the factors affecting the local blood donor’s satisfaction. While different ethnics had unique cultural identities, the general Malaysian population understood and communicated well in the Malay language, which is the national language of Malaysia. This study aimed to develop a Malay language questionnaire which measures blood donor’s satisfaction in blood centre situated within a tertiary hospital in Malaysia.

## Methods

This was a cross sectional study conducted among blood donors at the blood bank of Hospital Raja Permaisuri Bainun. All blood donors who completed donation and understand the Malay language were invited to participate in this study. Non-Malaysian and illiterate donors were excluded. The subjects were consecutively sampled, where every subject who arrived the blood donation centre and fulfilled the criteria of inclusion was selected. Data collection was conducted in July and August 2020. Informed consent was obtained from the participants before their participation. We had obtained the Medical Research and Ethics Committee, Ministry of Health Malaysia approval before study commencement.

### Stage 1: Questionnaire Framework Development

The questionnaire framework was developed initially through extensive literature review by the experts (consisting of transfusion medicine specialists and blood bank medical officers) using the MEDLINE database. The literatures published since inception until June 2020 were included into the search. The keywords chosen were blood donor, satisfaction, questionnaire, development and validation. Further search was undertaken by screening the bibliographies of the relevant articles found (7–10, 15, 16). The questionnaire was developed in the Malay language, the national language of Malaysia. Two rounds of experts meeting were then held to determine the conceptual framework, where the researchers identified the key elements contributed to the blood donors’ satisfaction.

### Stage 2: Content Validity

Content validity is defined as the adequacy of a questionnaire to assess the domain of interest (17). In the context of the current questionnaire, it is important to establish content relevance and clarity, so that the items can capture and represent the satisfaction level of the blood donors (18). The questionnaire developed in the previous stage was assessed by two content reviewers, that is, a transfusion medicine specialist and a senior science officer. Content validity was assessed based on the following criteria: appropriateness, comprehensibility and clarity of phrasing for all item (19).

### Stage 3: Cognitive Debriefing

Cognitive debriefing interviews are commonly used during questionnaire development to determine the face validity, the appropriateness of items and to assess participants’ understanding of the content of measures (20). Ten blood donors who visited the blood bank for blood donation were invited for this purpose. To ensure maximum variation of sample, we used purposive sampling in order to obtain the widest possible range of donors attending the blood bank (21). The investigators interviewed the donors to check the relevance, clarity and comprehensibility of items (22). The donors’ feedback was discussed among the investigators and revision in wording were made whenever it was deemed appropriate. The investigators agreed and reached consensus before the revised blood donor satisfaction questionnaire disseminated for field testing.

### Stage 4: Psychometric Testing

To ensure good psychometric properties of a questionnaire, it is important to evaluate the reliability and validity of the tool (23). Field testing is an established method to determine the reliability and validity of a new tool (24). The sample size for the field testing was calculated based on a ratio of 1 item:5 respondents (25). Our tool consisted of 18 items, hence we required a minimum sample size of 90. To account for 40% drop-out rate, the sample size was inflated to 150.

We employed the test-retest method to assess the stability of the questionnaire (17). During the field testing, eligible donors presented at the blood bank were invited to participate. Upon donor’s consent, participants were given two sets of identical, printed questionnaires. The donors were asked to complete the first set of questionnaires on the spot after the blood donation. The second set of questionnaires were given to the donors to bring home. The donors were required to fill up the second set of the questionnaire after two weeks, scan and send the completed questionnaire to the investigators by using the most commonly used social mobile application locally, i.e., WhatsApp.

## Construct Validity

Factor analysis was commonly used to determine the appropriate number of domains and whether the items fit the particular construct (17). We evaluated the construct validity using exploratory factor analysis. The component of the questionnaire was extracted using the principal component analysis and varimax rotation. Domains with eigenvalues exceeding one were retained (26). A low factor loading indicate that the items did not fit the construct. Hence, we retained items with factor loadings of 0.40 and above (27). Kaiser-Meyer-Olin (KMO) measure of sampling adequacy value of > 0.8 indicates sampling sufficiency, while a significant Barlett’s Test of the Sphericity (*P* < 0.001) indicated that the samples were suitable for factor analysis (26). IBM SPSS Statistics version 24.0 (IBM Corporation, New York, USA) was used to perform the data analysis.

## Reliability

Cronbach’s alpha assesses the internal consistency of the items within a questionnaire and describes the extent of variation for a set of items in the scale (28). A Cronbach alpha value of more than 0.7 indicated acceptable reliability (17). Reliability was also assessed using test-retest method. One-way random effects model with single measures was used in our model, to generate the intraclass correlation coefficient (ICC) value. An ICC values of 0.4–0.75 was considered as fair, while an ICC value ≥ 0.75 was considered as excellent (26).

## Results

### Stage 1: Questionnaire Framework

Based on a similar study by Trovão et al. (15), the initial questionnaire was developed and consisted of three domains, which were technical aspects [items 1, 2 and 3]; interpersonal aspects [items 4, 5, 6, 7 and 8] and accessibility/ convenience (items 12, 13, 15 and 16). Further literature search revealed that donors’ physical experience may affect their satisfaction level (7). Hence, the fourth domain of physical experience (items 9, 10 and 11) was added to the questionnaire. It is also noteworthy to understand the overall satisfaction and the intention of future donation among the blood donors (8). The overall satisfaction is important to prevent possible response bias due to the existing items (15). The fifth domain of overall satisfaction [items 17 and 18) was, therefore, added. The instrument consisted of a total of 17 items at this stage.

### Stage 2: Content Validity

Two content experts reviewed and assessed the content validity of the instrument. The first reviewer commented on the clarity of item number 1: *I am satisfied with the counter service*, and suggested to specify whether it means registration counter. The same reviewer also commented for item number 4: *I am satisfied with the service given by the interviewer*, whether the term ‘interviewer’ should be replaced with medical officers, as the interview was routinely performed by the medical officer.

The reviewer also commented on the wording of item number 3: withdraw blood (‘mengutip darah’), and suggested to replace it with ‘mengambil darah’. The word ‘mengutip’ and ‘mengambil’ had similar meaning in the Malay language. Upon consensus, we felt that the latter was more appropriate in the blood taking context, and hence we adopted the word ‘mengambil’.

In term of comprehensiveness of the questionnaire, one reviewer suggested to include the question *I am satisfied with the location of the blood donation centre*. While a strategic location of the blood donation centre is important, it is not feasible to shift the current blood donation centre to another location. Hence, the investigators decided not to include this question in the instrument, as it would not offer much value in service improvement.

At the previous stage, most literatures did not include car park in the blood donor satisfaction questionnaire. One content reviewer felt that adequate car park for blood donors is important to motivate them to come for donation. The investigators discussed and agreed that it was within the means of the hospital management in this regard. Hence, this question was included as item 14 in the questionnaire. Apart from the aforementioned, both content reviewers rated the appropriateness, comprehensibility and clarity of phrasing for all the item as satisfactory. The instrument consisted of 18 items at the end of this stage.

### Stage 3: Cognitive Debriefing

Ten donors were invited at the cognitive debriefing stage, in which they were interviewed in-depth regarding the relevance, clarity and comprehensibility of the instrument. The questionnaires were generally well accepted and understood by the respondents.

Four respondents commented on the word of choice for item number 13*: I am satisfied with the refreshment* [Malay: ‘pesegaran’] *offered after blood donation*. While the Malay word *pesegaran* literally means refreshment, the respondents found that the word is not relevant and hard to understand. It was suggested to use the more common word, food [Malay: ‘makanan’] to ensure clarity. Hence, the original phrasing of the item was revised, and changed to: *The food provided after blood donation are satisfactory*.

Two respondents doubted on the duplication of questions, which are items number 15 and 16. The phrase *I am satisfied with the waiting time before blood donation* and *I am satisfied with the overall process of blood donation* was entirely different domains but appeared similar in the perspective of donors. Since the two questions came in sequence in the paper-based questionnaire, it may cause confusion to the readers. Hence, we have decided to bold-size the word ‘waiting time’ and ‘overall process’ to enhance clarity.

Regarding the comprehensibility of the questionnaire, some respondents suggested to add Wi-Fi access and experienced phlebotomist as additional item in the instrument. However, we felt that these were beyond the normal scope of service provided by blood banks in the local public hospitals and hence should only be considered in more specific studies in the future.

### Stage 4: Psychometric Testing

Of the 160 invited blood donors, 23 (14.4%) refused to participate and 137 (85.6%) blood donors responded in the first phase. After two weeks, 101 (63.1%) responded to the questionnaire. Among those who had completed the two phases, majority were male (82, 81.2%), Malay (65, 64.4%), married (68, 67.3%), regular donors (75, 74.3%), with a mean age of 37.4 ± 10.76 years old (Table 1).

The KMO value for the psychometric field testing is 0.896 (> 0.8). The Bartlett’s Test of Sphericity is significant (*P* < 0.001), which signifies sampling adequacy and suitability for factor analysis. Variables with factor loadings more than 0.5 were considered as well loaded into a particular domain. Total of five domains were identified, with factor loadings ranging from moderate to high: technical domain (0.953–0.959); interpersonal domain (0.856–0.951); physical experience (0.902–0.931); accessibility and convenience (0.729–0.850); overall satisfaction (0.773–0.879). The items had satisfactory communalities value (> 0.3), ranging from 0.531–0.919, which signified that the extracted items represented the variables well.

Test-retest reliability represented the stability of the instrument. Out of the 137 respondents who completed the first phase of the questionnaire, 101 completed the second phase of retest. The Cronbach alpha internal consistency for all the domains was satisfactory, ranging from 0.814–0.955 (Table 2). The intraclass correlation coefficient of all the domains were excellent (0.771–0.847) except the overall satisfaction domain, which had a fair value of 0.663 (Table 3).

## Discussion

Based on our findings, the Malaysian blood donor satisfaction (M-DBS) questionnaire is a reliable and valid tool to measure donors’ satisfaction towards blood bank services. The Malay language questionnaire was developed based on extensive literature review, expert meetings, content review, cognitive debriefing of respondents and field testing to assess psychometric properties of the questionnaire, which are standard practices for questionnaire validation (17). Non-remunerated blood donors were the main source of blood procurement in Malaysia (13). Hence, a valid and reliable tool to assess blood donor’s satisfaction is necessary, as it serves as a timely feedback mechanism to enhance blood bank service quality and increase retention of non-remunerated blood donors in Malaysia.

The comprehensibility of the questionnaire was explored in the content review stage and the cognitive debriefing stage. The initial draft of the questionnaire consisted of five domains and 17 items. One content reviewer suggested to add in an item to explore the view of donors regarding the convenience of our current blood bank location. While the location of health care facilities is an important determinant of accessibility (30), our blood bank was located in the capital city of the Perak state and a relocation of the blood bank requires a large amount of resources and was not feasible in the near future.

The content reviewers subsequently proposed to include the car park item in the questionnaire. In developed countries like Germany, the adequacy of car park was considered as one of the components in the evaluation of donors’ satisfaction (31). In Brazil, a developing country, this item was not included as a satisfaction evaluation component (15), as this item may not be applicable to all the donors. Malaysia had the second highest ratio of registered road motor vehicles per 1,000 population in the Southeast Asia region (32). Hence, we reached consensus and agreed that car park is an important component to measure the satisfaction of the donors in the local context. We added this item in the finalised instrument, consisting of 5 domains and 18 items.

The field testing among 137 respondents demonstrated the M-BDS questionnaire as a tool with good construct validity. All the items had factor loadings of more than 0.70, indicating that the items were uniquely related and fitted to the particular domain (33). All the domains had good internal consistency, with Cronbach alpha values of more than 0.80 (28). The stability of the questionnaire was measured by test-retest, with intraclass correlation coefficient ranging from 0.665 to 0.837. The test-retest was conducted over a two-week period as suggested by previous literatures (26, 34).

Technical aspect, interpersonal aspect, physical experience, accessibility and convenience emerged as four major components which affect donors’ satisfaction. These domains echoed previous studies conducted in other countries (8–10, 15, 16.) Both the interpersonal aspects and accessibility/convenience were the domains with the most items in our instrument, comprising of five items each. All the items in the interpersonal domain had a factor loading of more than 0.9, except item number 4 with a factor loading of 0.856 (*I am satisfied with the service given by the interviewer*). In the accessibility and convenience domain, item number 14 had the lowest factor loading (*Car park location at blood donation centre is satisfactory*). Nevertheless, both the factor loadings were considered as satisfactory (> 0.4).

The physical experience of blood donors before and after blood donation and their perceived pain of venepuncture was found to be important predictors of their satisfaction (7). In a local study, it was reported that the fear of needle prick, pain or discomfort were major barriers for blood donation (29). During the cognitive debriefing, most donors agreed that the fear of needles may stop some people from donating blood. Within this context, all the item in the physical experience domain had high factor loading (0.902–0.931) and high Cronbach alpha (0.905). Hence, the physical experience domain was maintained in our questionnaire.

Apart from the four domains aforementioned, we had included the overall satisfaction domain in our questionnaire, which consisted of two items. Overall satisfaction is a key indicator in measuring the general satisfaction of the blood donors (8, 15). Nguyen et al. (8) reported that blood donor’s satisfaction was significantly associated with their intention to repeat blood donations. While the intent to return may not accurately predict the actual return for donation, this variable serves as an important marker for us to understand the factors which affects their intention to come for blood donation again. Both item in this domain had good factor loading (0.773–0.879) and good Cronbach alpha score (0.827). A lower ICC value of in this domain (0.663) may indicate the intention to donate blood again and the overall satisfaction of the donors may fluctuate over the two weeks period, which required further investigation.

The strength of this study lies in the absence of a local tool to evaluate the blood donors’ satisfaction. This is the first questionnaire of such, useful for the evaluation of donors’ satisfaction in blood bank and may be applicable in other blood banks nationwide. The M-BDS questionnaire was developed in the national language of Malaysia, the most widely used language in Malaysia and was socioculturally appropriate. The developed instrument had satisfactory psychometric properties, supported by good internal consistency and stability.

There are several limitations. The second test of the questionnaire was conducted at the respondents’ home, hence we could not ensure that they were answered by the intended person. Second, our sample size was based on an item-sample ratio of 1:5. Previous literatures suggested a ratio of 1:10 may increase the rigour of the study (17). Third, we only performed exploratory factor analysis without confirmatory factor analysis. To further confirm our findings, it is valuable for other researchers to validate the M-BDS questionnaire in a larger sample size of blood donors in other regions of Malaysia. Focus group discussion among the donors during the questionnaire development stage may provide additional information which are not conventionally known.

## Conclusion

The M-BDS questionnaire is a reliable and valid tool to measure blood donors’ satisfaction in Malaysia. Utilisation and revalidation of the instrument by other local blood donation centres may further confirm our findings.

## Figures and Tables

**Table 1:** Demographic characteristics of respondents (*n* = 137)

Characteristics	First phase *n* (%)	Second retest phase *n* (%)
Gender		
Male	108 (78.8)	82 (81.2)
Female	29 (21.2)	19 (18.8)
Age	37.5 ± 10.35	37.4 ± 10.76
Ethnicity		
Malay	91 (66.4)	65 (64.4)
Chinese	32 (23.4)	26 (25.7)
Indian	13 (9.5)	9 (8.9)
Others	1 (0.7)	1 (1.0)
Marital status		
Single	36 (26.3)	29 (28.7)
Married	94 (68.6)	68 (67.3)
Divorced	7 (5.1)	4 (4.0)
Occupation		
Government	44 (32.2)	32 (31.7)
Private	41 (29.9)	26 (25.7)
Self-employed	31 (22.6)	23 (22.8)
Student	14 (10.2)	13 (12.9)
Unemployed	7 (5.1)	7 (6.9)
Educational level		
Degree or advanced degree	40 (29.2)	36 (35.6)
Certificate or diploma	46 (33.6)	29 (28.7)
Secondary	50 (36.5)	35 (34.7)
No formal education	1 (0.7)	1 (1.0)
Income		
< RM2,000	40 (29.2)	23 (22.8)
RM2,000–RM4,000	56 (40.9)	43 (42.5)
RM4,001–RM8,000	25 (18.2)	23 (22.8)
> RM8,000	16 (11.7)	12 (11.9)
Donor status		
First time donor	8 (5.8)	4 (4.0)
Regular donor	101 (73.8)	75 (74.2)
Lapsed donor	28 (20.4)	22 (21.8)

**Table 2:** Factor loading of items in the questionnaires

Domain and components	Final items	Factor loading					Communalities

		1	2	3	4	5	
Technical	1. I am satisfied with the counter service	0.958					0.918
	2. I am satisfied with the service given before blood donation process (fill-up forms, haemoglobin measurement and body weight)	0.953					0.908
	3. I am satisfied with the skill of the staff who took my blood	0.959					0.919
Interpersonal	4. I am satisfied with the service given by the interviewer		0.856				0.732
	5. The staff on duty are friendly and polite		0.953				0.908
	6. The staff on duty are always ready to listen/help me		0.941				0.886
	7. After donating blood, the staff on duty thanks me affectionately		0.917				0.840
	8. The staff on duty communicated clearly with me over the blood donation process		0.951				0.904
Physical experience	9. Before donating blood, I felt healthy			0.931			0.866
	10. Blood donation is smooth and painless			0.902			0.813
	11. After donating blood, I felt healthy			0.920			0.846
Accessibility and convenience	12. I am satisfied with the cleanliness at the blood donation area				0.850		0.723
	13. The food provided after blood donation was satisfactory				0.843		0.711
	14. Car park location at blood donation centre is satisfactory				0.729		0.531
	15. I am satisfied with the operating hours for blood donation				0.770		0.592
	16. I am satisfied with the waiting time before blood donation				0.739		0.546
Overall satisfaction	17. I am satisfied with the overall process of blood donation					0.879	0.857
	18. I will donate blood again in the future					0.773	0.711

Notes: Extraction methods: principal component analysis; Rotation method: Varimax with Kaiser normalisation; Total variance explained was 86.36%

**Table 3:** Reliability of the questionnaires

Domain and components	Final items	Cronbach alpha	Corrected item-total correlation	Cronbach alpha if item deleted	Intraclass correlation coefficient (*n* = 101)
Technical	1. I am satisfied with the counter service	0.953	0.905	0.929	0.771
	2. I am satisfied with the service given before blood donation process (fill-up forms, haemoglobin measurement and body weight)		0.894	0.936	
	3. I am satisfied with the skill of the staff who took my blood		0.907	0.926	
Interpersonal	4. I am satisfied with the service given by the interviewer	0.955	0.784	0.962	0.779
	5. The staff on duty are friendly and polite		0.920	0.937	
	6. The staff on duty are always ready to listen/help me		0.901	0.940	
	7. After donating blood, the staff on duty thanks me affectionately		0.866	0.946	
	8. The staff on duty communicated clearly with me over the blood donation process		0.919	0.938	
Physical experience	9. Before donating blood, I felt healthy	0.905	0.839	0.841	0.763
	10. Blood donation is smooth and painless		0.782	0.886	
	11. After donating blood, I felt healthy		0.816	0.862	
Accessibility and convenience	12. I am satisfied with the cleanliness at the blood donation area	0.814	0.746	0.734	0.847
	13. The food provided after blood donation was satisfactory		0.739	0.741	
	14. Car park location at blood donation centre is satisfactory		0.593	0.827	
	15. I am satisfied with the operating hours for blood donation		0.586	0.787	
	16. I am satisfied with the waiting time before blood donation		0.564	0.801	
Overall satisfaction	17. I am satisfied with the overall process of blood donation	0.827	0.832	0.946	0.663
	18. I will donate blood again in the future		0.711	0.948	
